# Supplementary material for: The molecular basis of pyrazinamide activity on Mycobacterium tuberculosis PanD
Source: Nat Commun. 2020 Jan 17;11:339. doi: 10.1038/s41467-019-14238-3 (PMC6969166; doi:10.1038/s41467-019-14238-3)
Supplement: Supplementary file 1 — Supplementary Information [file 41467_2019_14238_MOESM1_ESM.pdf]

Supplementary Information

The molecular basis of Pyrazinamide activity on *Mycobacterium tuberculosis* PanD

Q.Sun *et al*

Supplementary Note 1

The apo *Mtb* PanD crystals are in the $P4_12_12$ space group and diffracted to 2.7 Å. These crystals were soaked in cryo-protectant with 10 mM POA . The structure was solved with molecular replacement using the *Mtb* PanD structure (pdb 2C45). After manual model building and refinement, the Rfactor and Rfree (%) are 20.30 /24.88 and 19.92/24.78 for both apo and POA-complex structures, respectively (**Supplementary Table 1**).

There are six copies of *Mtb* PanD in one asymmetric unit. The electron density of the active site residues indicated that all the PanD molecules in the crystal are cleaved and processed into separate β and α chain, as there was clear density for the pyrovyl modification and the cleavage between resdiues Gly24 and Ser25. There was no electron density observed for the last 24 amino acid residues (Asp116 – Gly139) and the C-terminal His-tag. Thus, each copy contains the β chain (Met1-Gly24) and α chain (Pyr25-Ile115).

The 6-Cl-POA complex crystal was prepared by co-crystallizing *Mtb* PanD protein in presence of 20 mM 6-Cl-POA. The crystals were in the $P3_1$ space group and diffracted to 2.25 Å resolution. The structure was obtained using molecular replacement. It was refined to an Rwork/Rfree (%) of 13.39/16.43. There are 12 copies of PanD protein in one asymmetric unit, which form three tetramers. The structures are very similar with an rmsd of the c-alpha between these tetramers are 0.24 Å, 0.37 Å, and 0.35 Å. The first tetramer (chains A-H) was used in the structure description.

The PanD mutant M117I was crystallized in the I422 space group. The crystal diffracted to 2.33 Å resolution. The structure was refined to Rwork/Rfree (%) as 17.43/20.38. There is a single PanD in one asymmetric unit, and the tetramer is formed through crystallographic symmetry. The model comprises the first 126 residues. When M117I was superimposed onto wild-type protein, the rmsd for Cα is below 0.4 Å. The major difference in the structure occurred at the C-terminus of the short peptide (His21-Gly24). It may reflect the internal flexibility of this region.

Supplementary Table 1. X-ray diffraction data collection and model refinement statistics †

	Mtb PanD (apo)	POA:PanD	6-Cl-POA:PanD	M117I PanD
**Data collection**				
Space group	$P\ 4_1\ 2_1\ 2$	$P\ 4_1\ 2_1\ 2$	$P\ 3_1$	I 422
Cell dimensions				
a, *b*, *c* (Å)	163.0, 163.0, 63.6	162.3, 162.3, 62.8	143.7, 143.7,59.8	73.0, 73.0,109.5
α, β, γ (°)	90, 90, 90	90, 90, 90	90, 90, 120	90,90,90
Resolution (Å)	21.92 - 2.7 (2.797 - 2.7)*	21.75 - 2.7 (2.796 - 2.7)	21.89 - 2.25 (2.33 - 2.25)	36.51 - 2.33 (2.414 - 2.33)
R_{sym} or R_{merge}	0.104 (1.108)	0.140 (1.455)	0.114 (0.545)	0.151 (0.495)
$I / \sigma I$	15.70 (2.08)	16.48 (2.31)	8.03 (1.90)	18.88 (3.75)
Completeness (%)	99.60 (98.85)	99.70 (99.87)	99.53 (96.47)	99.58 (96.95)
Redundancy	10.4 (10.8)	15.7 (15.5)	5.7 (4.9)	11.3 (5.4)
**Refinement**				
Resolution (Å)	21.92 - 2.7 (2.797 - 2.7)	21.75 - 2.7 (2.796 - 2.7)	21.89 - 2.25 (2.33 - 2.25)	36.51 - 2.33 (2.414 - 2.33)
No.reflections (total/unique)	249355 /24034	369329/23578	373985/65250	74868/6633
R_{work} / R_{free} (%)	20.30 /24.88	19.92/24.78	13.39/16.43	17.43/20.38
No. atoms	5247	5296	10543	1002
Protein	5194	5203	10356	947
Ligand/ion	30	84	180	16
Water	23	9	7	39
B-factors (Å2)				
Protein	69.4	61.3	55.48	38.96
Ligand/ion	75.2	64.4	59.80	61.26
Water	41.8	39.0	45.93	43.43
R.m.s. deviations				
Bond lengths (Å)	0.013	0.013	0.015	0.006
Bond angles (°)	1.68	1.68	1.80	0.92
Ramachandran				
Favoured (%)	98.19	99.55	99.77	98.35
allowed (%)	1.81	0.45	0.23	1.65

† The table was generated with Phenix "Table 1" function.

*Values in parentheses are for highest-resolution shell.

Supplementary Table 2. Primers used for mutagenesis

Primer	Sequence
H21R-f	ccgacctgcgcTACGTCGGCTCGGTGACC
H21R-r	agccgacgtagcgCAGGTCGGCGCAGG
M117I-f	aaaccgatcgacataGGCCACGATCCGGCATTTGTG
M117I-r	atcgtggcctatGTCGATCGGTTTGTTGTAAGCG

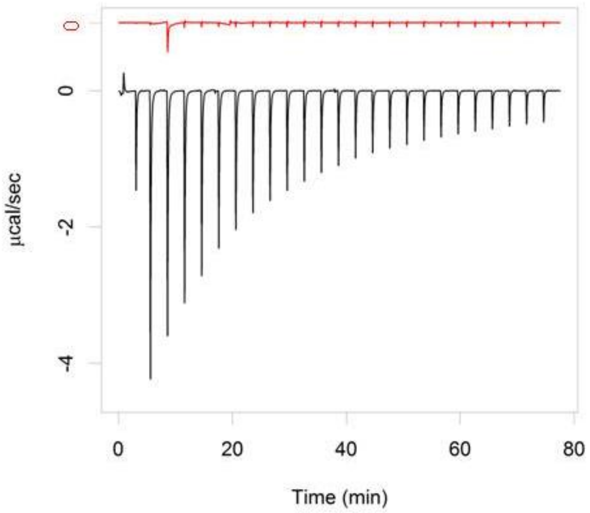

Supplementary Figure 1. Isothermal titration calorimetry result for titration of POA into *Mtb* PanD. The black trace shows the heat released from the titration of 20 mM POA against 0.5 mM *Mtb* PanD in 100 mM Tris pH 7.5. In red curve is the titration of 20 mM POA in 100 mM Tris buffer pH 7.5 in the same scale of the POA-to-PanD curve. The POA stock was also in 100 mM Tris pH 7.5. From five separate experiments, it was calculated that K_d = 0.71 (0.03) mM, ΔH = -4200 (200) cal/mol, ΔS = 0.1 (0.7) cal/mol/deg

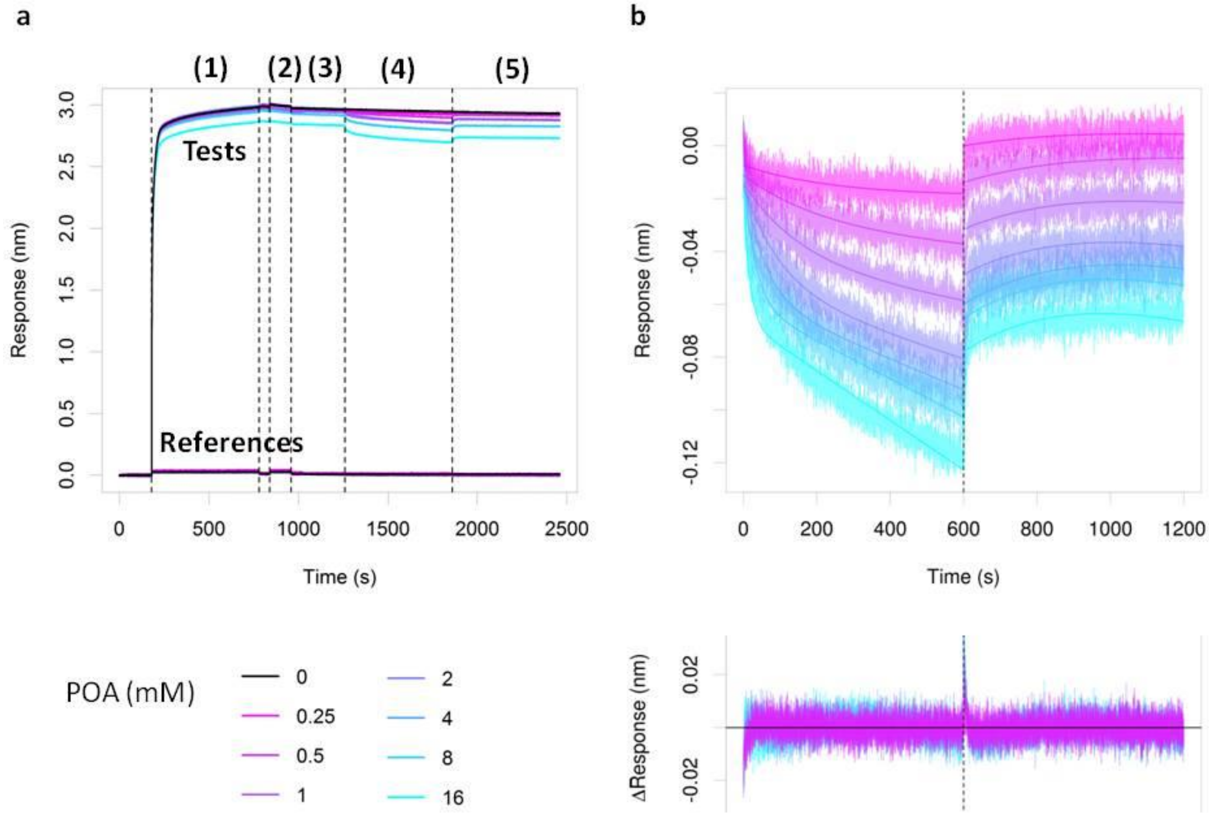

Supplementary Figure 2. POA binds to *Mtb* PanD slowly but tightly. a. Raw data of a typical BLI run. The top 8 curves were the raw data with the test biosensors loaded with biotinylated *Mtb* PanD; the bottom 8 overlapped curves were signals from reference biosensors loaded with biocytin instead. The critical steps are labeled: (1) Load, (2) Block, (3) Baseline, (4) Association, (5) Dissociation. Details are described in **Methods**. b. Fitting of association-then-dissociation of the BLI data. The top panel is the processed BLI sensorgram of *Mtb* PanD binding to POA. The residuals from the fitting are shown in the bottom panel. k_{on} was measured in triplicate as 3.5 (0.6) $M^{-1}s^{-1}$, k_{off} as 0.0027 (0.0001) s^{-1}. K_d was derived as 0.8 (0.1) mM.

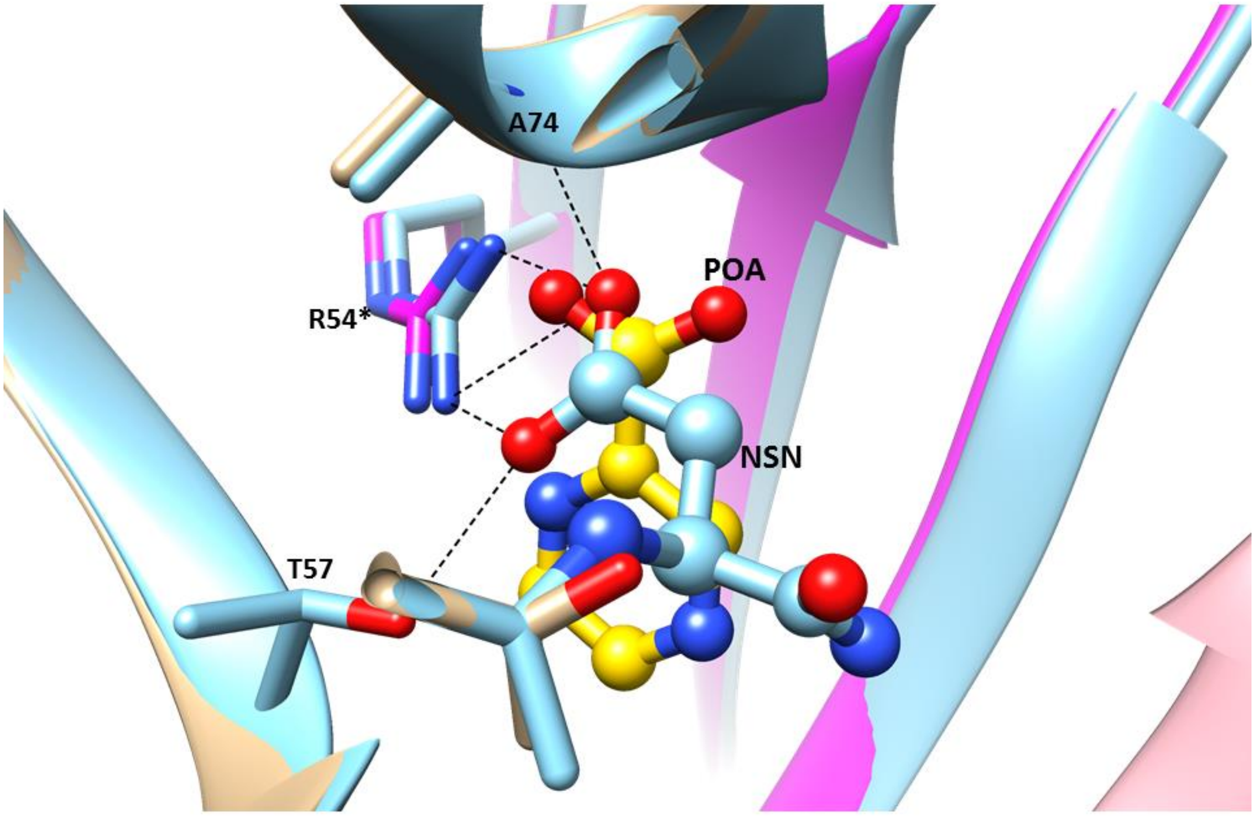

Supplementary Figure 3. POA binds to the PanD active site. The two subunits of *Mtb* PanD tetramer are shown in tan and magenta ribbons, respectively. POA is located in the active site at the interface of these two subunits, and is shown with carbon atoms in yellow. Superimposed is the crystal structure of *H. pylori* PanD with isoasparagine (NSN), a substrate analog (pdb 1UHE). *H. pylori* PanD is shown in cyan. Both NSN and POA are in ball-and-stick. The hydrogen bond with NSN are shown as dashes, Arg54*, Thr57 and Ala74.
